# INTERPROFESSIONAL CO-LEARNING AT MULTIPLE LEVELS OF LTSS: PRO-HOME PROGRAMS AND RESEARCH

**DOI:** 10.1093/geroni/igad104.1807

**Published:** 2023-12-21

**Authors:** Jordan Skowronski, Joseph Zanoni, Maria Caceres

**Affiliations:** University of Illinois Chicago, Chicago, Illinois, United States; University of Illinois Chicago, Chicago, Illinois, United States; University of Illinois Chicago, Chicago, Illinois, United States

## Abstract

Promoting Seniors’ Health with Home Care Aides (Pro-Home), a randomized controlled trial (RCT) of a gentle physical activity program delivered by home care aides (HCAs) for their older clients in English or Spanish, provided unprecedented opportunities for collaboration and co-learning across multiple disciplines and professions (public health, health services research, sociology, medicine, physical/occupational therapy, kinesiology, social work, psychology, economics, medicine, nursing; frontline care workers, leaders and staff members of state and home care agencies, , social service agencies, researchers, undergraduate and graduate students). The turbulent long-term services and supports (LTSS) environment, which culminated in the COVID-19 pandemic, provided the study participants (HCAs and their older clients), multiple levels of LTSS stakeholders and the research team with rapidly evolving new contexts for the RCT. This paper presents how the Pro-Home project developed into a learning organization where individuals and organizations learn from each other to pivot at every new turn in the environment, focusing on the development of a novel interactive training for HCAs in-person or remotely. The initial training activities were conducted in-person. After adapting the material for remote delivery, we provided the latter sessions via phone with digital and physical tools. For each training session, we assessed outcome quality using an exit survey and other methods capturing HCAs’ program delivery. The presentation concludes with implications for research and practice in LTSS.

